# Achieving Self-Supported Hierarchical Cu(OH)_2_/Nickel–Cobalt Sulfide Electrode for Electrochemical Energy Storage

**DOI:** 10.3390/mi14010125

**Published:** 2023-01-02

**Authors:** Sa Lv, Wenshi Shang, Yaodan Chi, Huan Wang, Xuefeng Chu, Boqi Wu, Peiyu Geng, Chao Wang, Jia Yang, Zhifei Cheng, Xiaotian Yang

**Affiliations:** Key Laboratory for Comprehensive Energy Saving of Cold Regions Architecture of Ministry of Education, Jilin Jianzhu University, Changchun 130118, China

**Keywords:** hierarchical structure, copper foam, electrodeposition, electrochemical energy storage

## Abstract

Herein, nickel–cobalt sulfide (NCS) nanoflakes covering the surface of Cu(OH)_2_ nanorods were achieved by a facile two-step electrodeposition strategy. The effect of CH_4_N_2_S concentration on formation mechanism and electrochemical behavior is investigated and optimized. Thanks to the synergistic effect of the selected composite components, the Cu(OH)_2_/NCS composite electrode can deliver a high areal specific capacitance (*C*s) of 7.80 F cm^−2^ at 2 mA cm^−2^ and sustain 5.74 F cm^−2^ at 40 mA cm^−2^. In addition, coulombic efficiency was up to 84.30% and cyclic stability remained 82.93% within 5000 cycles at 40 mA cm^−2^. This innovative work provides an effective strategy for the design and construction of hierarchical composite electrodes for the development of energy storage devices.

## 1. Introduction

In recent years, transition metal sulfides (TMS) as electrode materials have continued to attract attention in the field of energy storage [1]. Among them, nickel sulfide and cobalt sulfide have special advantages in redox activity and theoretical specific capacitance (Cs). At the same time, they exhibit controllable morphology, abundant reserves, and environmental friendliness [2]. Various synthetic pathways have been explored to regulate their morphology and structure to achieve performance optimization, including hydrothermal, ion exchange, and electrodeposition methods [3,4,5]. Among them, electrodeposition technology has attracted continuous attention due to its simple operation, short experimental cycle, and easy realization of effective regulation of deposition amount [6,7]. In particular, the in situ electrodeposition based on the electrode substrate allows the binder-free electrode to be measured directly [8]. 

At the same time, researchers also optimized electrode performance by constructing nickel (cobalt) sulfide-based composite electrodes, including designing a bimetallic sulfide electrode. For example, He et al. fabricated hierarchical Ni-Co-S (NCS) electrodes using an electrodeposition method, and the Cs of the optimized electrode reached 640 F g^−1^ at 1 A g^−1^ and cyclic stability remained 84% within 10,000 cycles. The outstanding energy storage behavior is attributed to the hollow nanosphere structure, which is composed of interconnected NCS nanosheets and thus provides a smooth path for ion transport [9]. In another example, Zheng et al. reported a self-supported hierarchical NCS nanosheet by a two-step hydrothermal that exhibits ultra-high Cs [3]. In fact, the Cs value depends on the electrochemical active site of the constructed NCS electrode. Therefore, optimizing energy storage properties could be achieved by increasing the quality of deposited NCS electrode material [10,11]. However, with the continuous increase of the amount of deposition layer, electrode materials are prone to excessive disorderly accumulation, and even structure collapse and shedding may occur during the electrochemical test, which will inevitably lead to the attenuation of Cs and cyclic stability of the electrode [2,9,12]. 

To address the above issues, the construction of NSC-based composite electrodes has proved to be an effective strategy [13,14]. For instance, Luo et al. grew NCS on carbon nanotubes by combining chemical vapor deposition with electrodeposition. The introduction of carbon nanotubes, as scaffolds, provides spatial support for the growth of NCS and improves the conductivity of the composite electrode, which further provides an excellent passage for electron transport, and thus dramatically enhances Cs [11]. 

Inspired by this, we designed and constructed a Cu(OH)_2_/NCS composition electrode using a facile two-step electrodeposition. First, copper foam (CF) was selected as the only copper source for surface oxidation to generate Cu(OH)_2_ nanorod arrays, and the electrodeposition of NCS on its surface was then completed. The morphology and properties of Cu(OH)_2_/NCS electrode were studied by adjusting CH_4_N_2_S concentration. The advantage of this in situ electrodeposition strategy is that the electrode to be measured is directly obtained, and this allows each component to form close contact, reduces contact resistance, and thus optimizes the electron transmission path. This is especially true for Cu(OH)_2_ nanorods introduced by electrodeposition, which provides sufficient space for NCS deposition, and thus endows the hierarchical Cu(OH)_2_/NCS electrode with more abundant electrochemical active sites and outstanding energy storage characteristics. 

## 2. Materials and Methods

### 2.1. Materials and Reagents

Cut the commercial copper foam (CF) according to the specified size (1 cm × 1.5 cm), and then use water, ethanol and hydrochloric acid to complete the pretreatment, and vacuum dry for standby. The reagents, including NiCl_2_·6H_2_O, Co(NO_3_)_2_·6H_2_O, CH_4_N_2_S, and NaOH are exploited without further purification. 

### 2.2. Electrodeposition of Hierarchical Cu(OH)_2_/NCS on CF 

Galvanostatic deposition of Cu(OH)_2_ is achieved at 0.05 A for 300 s through a three-electrode system consisting of CF (working electrode), saturated calomel electrode (SCE, reference electrode), and Pt plate (counter electrode). A 2 M NaOH solution is used as electrolyte. 

A similar three-electrode system, with the working electrode replaced by the previously generated Cu(OH)_2_/CF, is used to achieve potentiostatic deposition of the NCS layer at −1.1 V for 1200 s. The electrolyte is composed of 0.05 M NiCl_2_·6H_2_O, 0.05 M Co(NO_3_)_2_·6H_2_O and 0.75 M CH_4_N_2_S. For comparison, potentiostatic deposition of NCS was performed for CH_4_N_2_S at different concentrations, including 0.05 M (abbreviated as S-1), 0.25 M (S-2), 0.5 M (S-3), 0.75 M (S-4), and 1 M (S-5). 

### 2.3. Characterization 

Samples were evaluated by XRD (Cu Kα radiation with λ = 1.5406 Å), FE-SEM (JSM-7610F), and XPS (ESCALAB 250Xi). Electrochemical characteristics, including cyclic voltammetry (CV), galvanostatic charge-discharge (GCD), and cycling performance, were determined by an electrochemical workstation (Chenhua, CHI 760E). Cu(OH)_2_/NCS/CF (working electrode), Ag/AgCl (reference electrode), and Pt plate (counter electrode) in 2 M NaOH solution constituted the three-electrode test system.

## 3. Results

**Figure 1:**
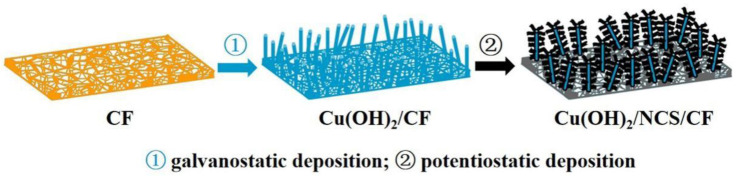
Synthesis path of hierarchical Cu(OH)_2_/NCS electrode.

Figure 1 is a schematic diagram of the synthesis path of the Cu(OH)_2_/NCS composite electrode by a facile two-step electrodeposition. The first step is to achieve surface oxidation to generate blue-green Cu(OH)_2_ nanorods on the orange-red 3D CF substrate by galvanostatic deposition, and then to achieve the encapsulation of Cu(OH)_2_ nanorods by NCS through potentiostatic deposition, thus forming the hierarchical Cu(OH)_2_/NCS composite electrode. 

**Figure 2:**
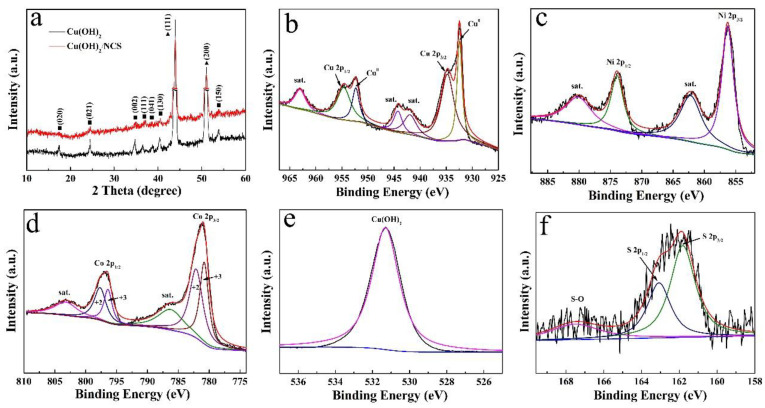
XRD patterns of Cu(OH)_2_ and Cu(OH)_2_/NCS (**a**). XPS spectra of Cu 2p (**b**), Ni 2p (**c**), Co 2p (**d**), O 1s (**e**), and S 2p (**f**).

Figure 2a illustrates the XRD spectra of Cu(OH)_2_ and Cu(OH)_2_/NCS. Two obvious strong peaks marked by a triangle are ascribed to the CF substrate (JCPDS No.01-1241), while the other seven peaks marked by a square are indexed to (020), (021), (002), (111), (041), (130), and (150) planes of orthorhombic Cu(OH)_2_ (JCPDS No.13-0420). No significant additional diffraction peaks are obtained in the XRD pattern of NCS due to the weak crystallinity of the NCS layer. This situation has been mentioned and explained in previous reports [4,15,16]. In addition, the XPS survey spectra of Cu(OH)_2_/NCS confirmed the presence of Cu, Ni, Co, O and S elements in the sample. Figure 2b records the spectrum of Cu 2p; two main peaks at 954.50 and 934.70 eV could be assigned to Cu 2p_1/2_ and Cu 2p_3/2_, while two separated sharp diffraction peaks at 952.45 and 932.50 eV originate from the CF substrate. The rests at 963.04, 944.80, and 941.94 eV are their satellite peaks [17,18]. The Ni 2p spectrum is depicted in Figure 2c. Two intense feature peaks at 873.80 and 856.20 eV are ascribed to Ni 2p_1/2_ and Ni 2p_3/2_, implying the presence of Ni^2+^. The other two peaks located at 880.00 and 861.80 eV are their corresponding satellite peaks [5,19]. In the Co 2p spectrum (Figure 2d), spin-orbit double peaks appeared at 797.60 and 796.40 eV indexed to Co 2p_1/2_, while those at 782.10 and 780.80 eV belong to Co 2p_3/2_, accompanied by two satellite peaks around 803.55 and 786.85 eV, confirming the coexistence of Co^2+^ and Co^3+^ [4,11]. For the O 1s spectrum (Figure 2e), the only characteristic peak at 531.20 eV is indexed to the characteristic signatures of Cu(OH)_2_ [8]. With respect to the S 2p spectrum in Figure 2f, the main peak decomposed into two peaks at 161.75 and 163.00 eV, corresponding to S 2p_1/2_ and S 2p_3/2_, respectively, and derived from S^2-^. At the same time, a weak broad peak at 167.40 eV could be attributed to the S-O bond. Oxygen is derived from OH^-^ generated by hydrolysis of CH_4_N_2_S, which is in good agreement with previous reports [19,20].

**Figure 3:**
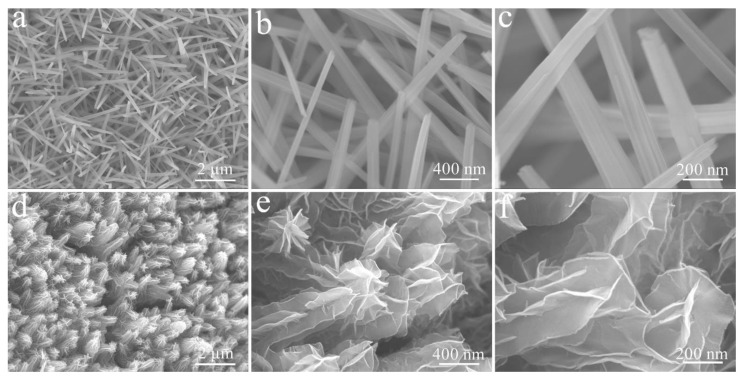
FE-SEM images of Cu(OH)_2_ (**a**–**c**) and Cu(OH)_2_/NCS (**b**–**f**) at different magnifications.

Figure 3a–c shows SEM images of Cu(OH)_2_ grown on CF by galvanostatic deposition at different magnifications. These Cu(OH)_2_ nanorods are densely and evenly coated on the surface of CF with a diameter of ca. 125 nm and a smooth surface. The corresponding Cu(OH)_2/_NCS formed by further potentiostatic deposition treatment is shown in Figure 3d–f. These Cu(OH)_2_ nanorods become fluffy due to the uniform coverage of NCS. In fact, these nanorods are tightly wrapped and wound by large curved nanoflakes with a thickness of ca. 8 nm. Therefore, the top of the nanorods form a flower-like structure. 

**Figure 4:**
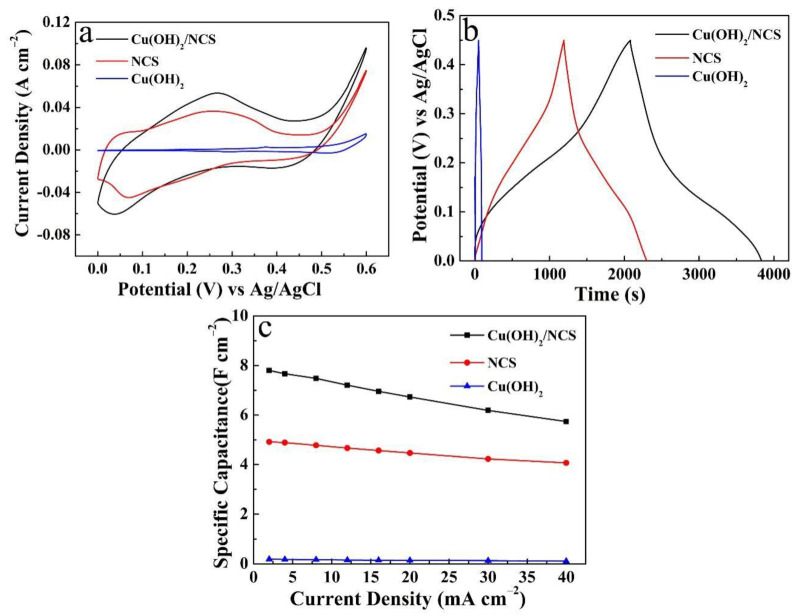
Performances comparison of Cu(OH)_2_/NCS, NCS, and Cu(OH)_2_ electrode: (**a**) CV curves; (**b**) GCD curves at different current densities and corresponding *C*s (**c**).

The electrochemical behaviors of the three electrodes were further tested and compared, including Cu(OH)_2_/NCS, NCS, and Cu(OH)_2_ electrode. Figure 4a presents the CV curves of the three electrodes at a scan rate of 5 mV s^−1^. It is evident that the Cu(OH)_2_/NCS electrode was observed to have a maximum current response. According to the rule, under the same scan rate, the larger the area enclosed by the CV curve, the higher the *C*s [10]. Therefore, the Cu(OH)_2_/NCS electrode reflects the relatively optimal energy storage property, which is attributed to the following pseudocapacitance reactions [4,6]:

(1) $$\mathrm{NiS}\;+\;OH^{-}↔\mathrm{NiSOH}\;+\;e^{-}$$

(2) $$\mathrm{CoS}\;+\;OH^{-}↔\mathrm{CoSOH}\;+\;e^{-}$$

(3) $$\mathrm{CoSOH}\;+\;OH^{-}↔\mathrm{CoSO}\;+\;H_{2}O\;+\;e^{-}$$

The inference can also be reflected in the GCD curves of the three electrodes. As displayed in Figure 4b, the Cu(OH)_2_/NCS electrode obviously has the longest discharge time at a discharge current density of 2 mA cm^−2^. Figure 4c lists a comparison diagram of the *C*s of the three electrodes at different discharge current densities. It also confirms that the Cu(OH)_2_/NCS electrode has the largest *C*s, followed by the NCS electrode, while Cu(OH)_2_ is the lowest. Therefore, the introduction of Cu(OH)_2_ is a feasible strategy to significantly enhance the energy storage properties of the NCS electrode. In addition, the deposition amount of NCS was regulated by changing the CH_4_N_2_S concentration to optimize the performance of Cu(OH)_2_/NCS composite electrode.

**Figure 5:**
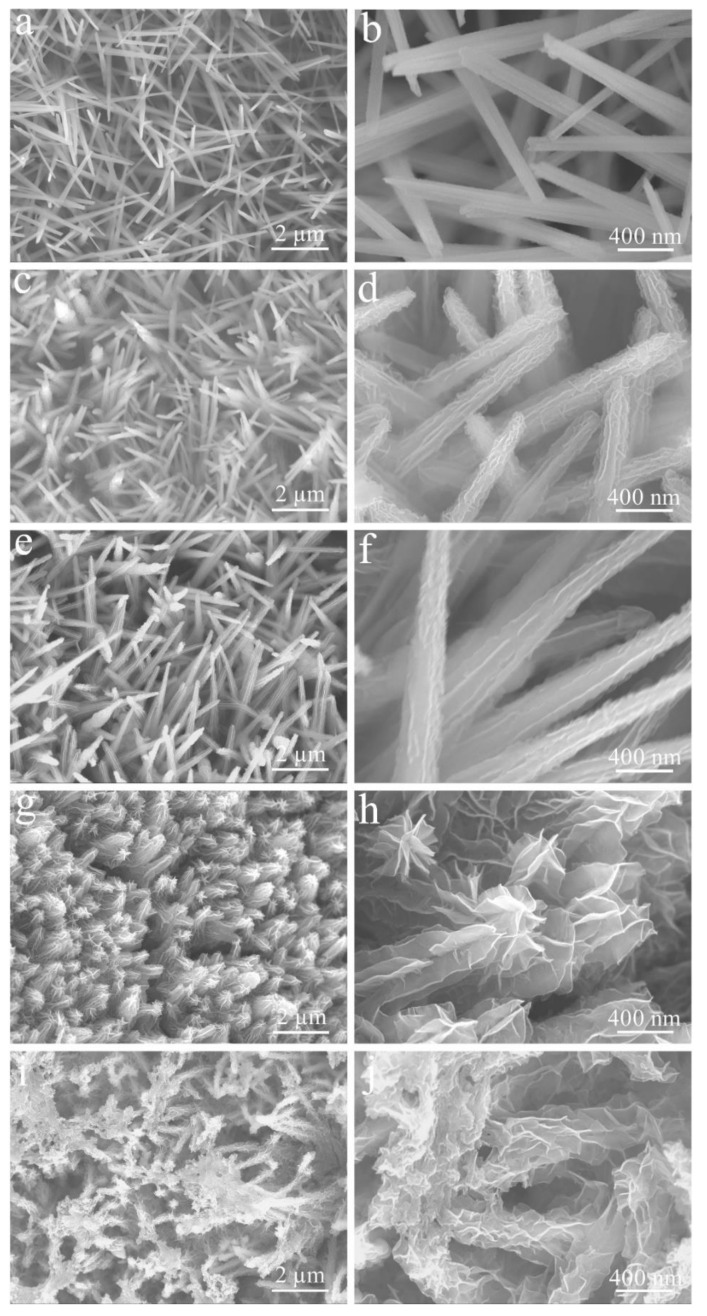
FE-SEM images of NCS electrodeposited on Cu(OH)_2_ nanorods for different CH_4_N_2_S concentrations: (**a**,**b**) 0.05 M, S-1; (**c**,**d**) 0.25 M, S-2; (**e**,**f**) 0.5 M, S-3; (**g**,**h**) 0.75 M, S-4; (**i**,**j**) 1 M, S-5.

Figure 5 displays the SEM image of the Cu(OH)_2_/NCS electrode deposits at different CH_4_N_2_S concentrations. When the concentration of CH_4_N_2_S was low (0.05 M, S-1), the surface of these Cu(OH)_2_ nanorods began to become rough, indicating a preliminary small deposition of NCS (Figure 5a,b). With the increase in CH_4_N_2_S concentration (0.25 M, S-2), it is evident that there are curved nanoflakes produced on the surface of these Cu(OH)_2_ nanorods, with a thickness of ca. 8 nm (Figure 5c,d). When the CH_4_N_2_S concentration increases to 0.5 M (S-3), these nanoflakes are connected to each other and arranged in rows, like ridges growing from nanorods (Figure 5e,f). When the concentration of CH_4_N_2_S reaches 0.75 M (S-4), these ridges gradually expand and rise, forming large undulating nanoflakes to wrap these Cu(OH)_2_ nanorods (Figure 5g,h). The concentration of CH_4_N_2_S continued to increase excessively (1 M, S-5), resulting in fragmentation and disordered accumulation of these nanoflakes (Figure 5i,j).

**Figure 6:**
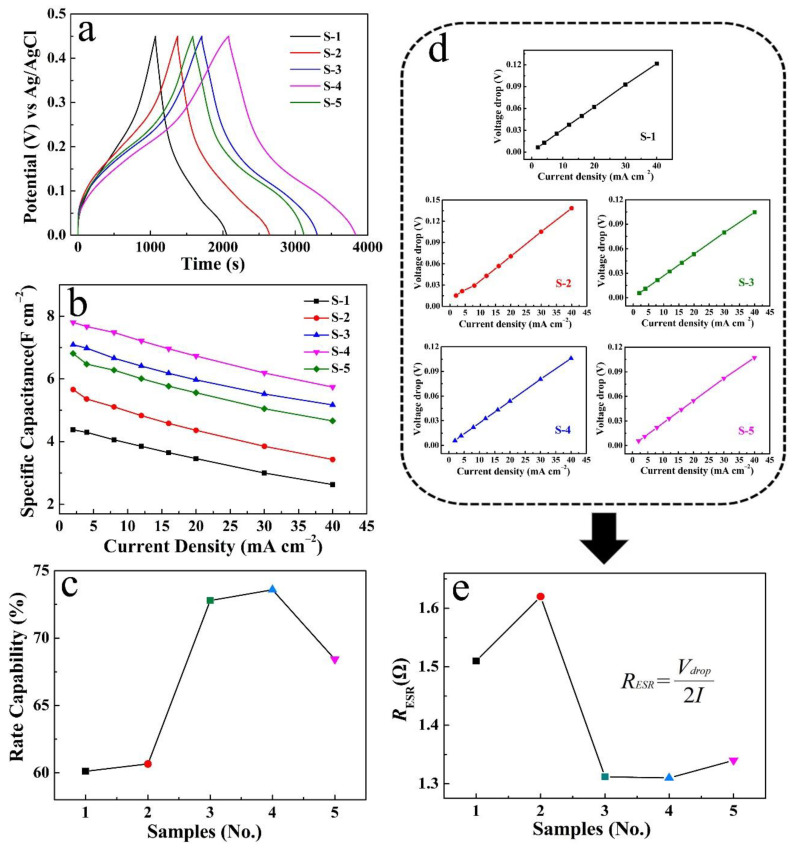
Performances comparison of S-1, 2, 3, 4 and 5: (**a**) GCD curves at different current densities, (**b**) corresponding *C*s and rate capability (**c**), (**d**) voltage drops and corresponding average *R*_ESR_ (**e**).

Figure 6 compares the electrochemical properties of the five electrodes. As seen, the GCD curves at current densities of 2 mA cm^−2^ (Figure 6a); the discharge time length sequence of the five electrodes is S-4 > S-3 > S-5 > S-2 > S-1, which also reflects the order of their *C*s. Further, their detailed values of *C*s corresponding to different current densities are shown in Figure 6b. It is clear that S-4 has the highest *C*s. As a further derivation, when the current density is increased by 20 times, S-4 also reflects a relatively optimal rate capability of 73% (Figure 6c). Figure 6d displays the voltage drop curves of the five electrodes at different current densities. Average *R*_ESR_ data derived from these are shown in Figure 6e. S-4 has a minimum *R*_ESR_ of 1.31 Ω cm^−2^ in terms of the formula inserted. 

According to the above comparative analysis, it can be inferred that in the case of Cu(OH)_2_ nanorods providing skeleton support, the *C*s value increases with the continuous deposition of NCS. At the beginning of the electrodeposition reaction, the S^2−^ produced by the gradual hydrolysis of CH_4_N_2_S is captured by Ni^2+^ and Co^2+^ ions, the formed NCS precipitated on the surface of Cu(OH)_2_ nanorods and acts as growth points [9]. The increase of CH_4_N_2_S concentration makes it provide more S^2−^, and thus the area of the deposited nanoflakes gradually increases; that is, the active area of the electrode increases [21]. However, the deposition of NCS nanoflakes is based on the premise that Cu(OH)_2_ nanorods provide skeleton support. When the amount of S^2−^ is too large, it is bound to collapse Cu(OH)_2_ nanorods and lead to a disordered accumulation of the NCS deposition layer [2,11]. In this case, excessive accumulation of the NCS layer leads to an increase in the average *R*_ESR_. Moreover, it is easy to fall off during electrochemical testing, resulting in reduced electrode rate capability [9]. Therefore, S-4 is considered an optimized structure for further consideration and systematic investigation. 

**Figure 7:**
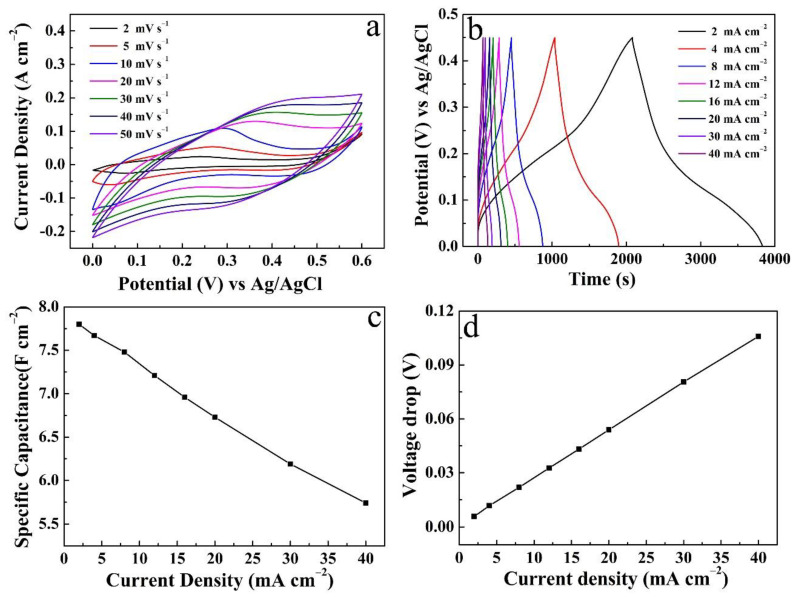
Electrochemical properties of Cu(OH)_2_/NCS electrode (S-4): (**a**) CV curves; (**b**) GCD curves at different current densities and corresponding *C*s (**c**) and the voltage drops (**d**).

**Figure 8:**
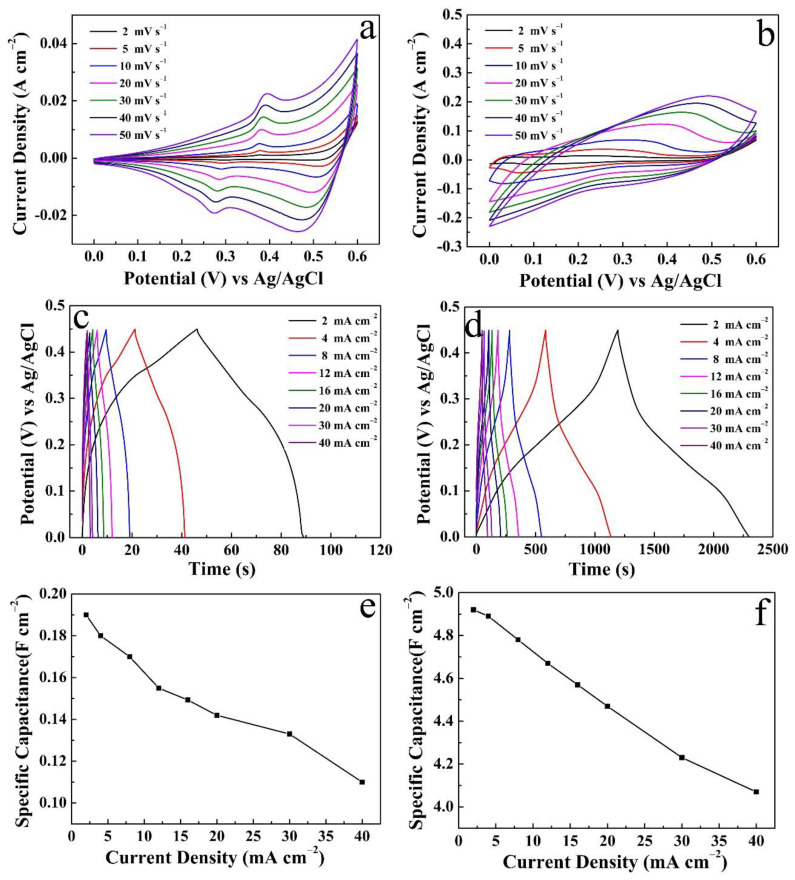
CV curves, GCD curves at different current densities and corresponding *C*s of Cu(OH)_2_ (**a**,**c**,**e**) and NCS electrode (**b**,**d**,**f**).

Figure 7a reveals the CV curves of the Cu(OH)_2_/NCS electrode (S-4) scan from 2 to 50 mV s^−1^. The increase of scan rate is accompanied by a gradual increase of the area enclosed by CV curves, while *C*s decrease gradually [17]. The smaller the scanning rate, the more the sufficient pseudocapacitance reaction occurs between OH^−^ from the electrolyte and Cu(OH)_2_/NCS electrode [11]. The GCD curves of the corresponding electrode at a discharge current density of 2–40 mA cm^−2^ are shown in Figure 7b. It is obvious that the smaller the current density, the longer the discharge time, and thus the greater the *C*s. Detailed *C*s values at different current densities are shown in Figure 7c. When the current density is 2 mA cm^−2^, the *C*s is up to 7.80 F cm^−2^, and the coulombic efficiency is up to 84.30% according to the formula [22,23]. Furthermore, the *C*s value remains 73.60% of the original when the current density increases by 20 times and reaches 40 mA cm^−2^. The average *R*_ESR_ derived from the voltage drop curve in Figure 7d is 1.31 Ω cm^−2^, which has been compared and discussed in Figure 6e. In addition, data from individual Cu(OH)_2_ and NCS electrodes were also processed (Figure 8), including CV curves, GCD curves at various current densities, and their corresponding *C*s.

**Figure 9:**
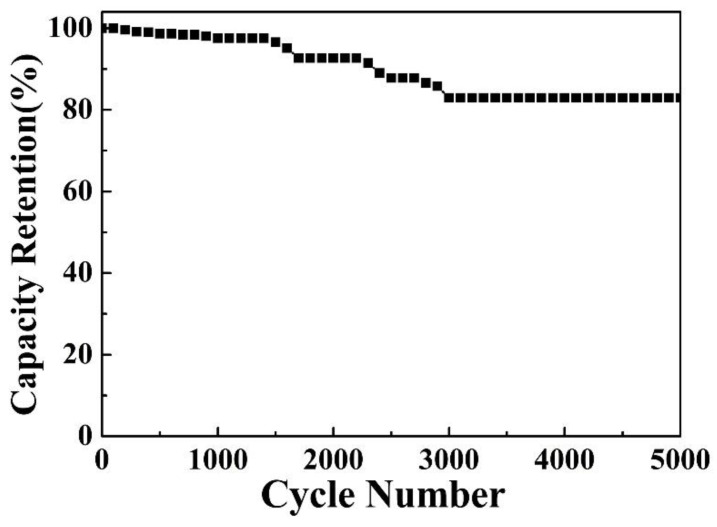
Cyclic stability of the Cu(OH)_2_/NCS electrode.

The Cu(OH)_2_/NCS electrode was further tested for long-term cycling stability in a three-electrode system at 40 mA cm^−2^ (Figure 9). The *C*s reached 92.68% of the original value at 2000 cycles, and still retained 82.93% within 5000 cycles. 

Table 1 lists the relevant Cu(OH)_2_ and TMS electrodes reported in recent literature.

The relatively superior properties of the Cu(OH)_2_/NCS electrode are ascribed to the following: (1) CF acts as the electrode substrate, which not only reflects the advantages of good electrical conductivity and 3D porous structure, but also serves as the only source of Cu in Cu(OH)_2_. The oxidation reaction achieved by the in situ electrodeposition is bound to result in uniform and dense growth of Cu(OH)_2_. In particular, the binder-free electrode to be measured can be obtained directly [7,10,17]; (2) the Cu(OH)_2_ nanorod arrays obtained, as skeleton support, provide sufficient growth space for deposition of the NCS layer, and the stretched NCS nanoflakes further expand the contact area between the Cu(OH)_2_/NCS electrode and the NaOH electrolyte, thereby creating more abundant electrochemical active sites [6,7,11]; (3) this hierarchical structure design provides a smoother path for charge transmission and thus effectively promotes the redox reaction process, while giving full play to the advantages of electrodeposition and the synergistic effect of each component, and obtaining excellent energy storage performance [21,28,30].

## 4. Conclusions

In summary, an electrodeposition path is presented to design a hierarchical Cu(OH)_2_/NCS composite electrode on CF. CF substrate acts as copper source and oxidizes to form Cu(OH)_2_ nanorods. The deposition parameters of NCS were further optimized by adjusting CH_4_N_2_S concentration. Benefiting from the synergistic effect of the selected composite components, the Cu(OH)_2_/NCS electrode delivers a high *C*s of 7.80 F cm^−2^ at 2 mA cm^−2^ and retains 5.74 F cm^−2^ at 40 mA cm^−2^, and excellent cyclic stability of 82.93% after 5000 cycles at 40 mA cm^−2^. Furthermore, the hierarchical structure design implemented by the in situ electrodeposition strategy provides an effective route for the construction of Cu(OH)_2_- or NCS-based composite electrodes for energy storage.

## Figures and Tables

**Table 1:** Comparison of *C*s between Cu(OH)_2_/NCS and literature reports.

Active Material	Substrate	Electrolyte	Current Density (mA cm^−2^)	*C*s (F cm^−2^)	Refs.
Cu(OH)_2_	Carbon cloth	1 M NaOH	1	0.24	[24]
Cu(OH)_2_	Cu foam	5 M NaOH	2	1.75	[8]
Co-Ni-S	Ni foam	3 M KOH	1	6.30	[25]
Cu(OH)_2_@MnO_2_	Cu foam	6 M KOH	2	0.71	[26]
Co(OH)_2_/CoOOH/ Co_3_O_4_/Cu(OH)_2_	Cu foam	1 M KOH	1	1.94	[27]
ZnNiFe-LDH/Cu(OH)_2_	Cu foam	6 M KOH	3	6.1	[28]
MoS_2_/Cu(OH)_2_	Carbon fiber paper	6 M KOH	1	1.12	[29]
NiMoO_4_@Ni-Co-S	Ni foam	2 M KOH	5	2.27	[6]
Cu(OH)_2_/Ni-Co-S	Cu foam	2 M NaOH	2	7.80	this work

## Data Availability

Not applicable.
